# Praiseworthy

**Published:** 1849-07

**Authors:** 


*Praiseworthy.*—Among the proceedings of the last regular meeting of the Pennsylvania Society of Dental Surgeons, is the following resolution, offered by Dr. J. D. White:

"*Resolved,* That a premium of twenty-five dollars, in a gold medal, be awarded by this Society, for the greatest improvement in porcelain teeth, viz. single gum-teeth, molar and bicuspid, plate and pivot teeth; twenty-five of each kind to be sent to either of the following Committee. Also,

"*Resolved,* That a medal of twenty-five dollars be awarded for the best block teeth that may be presented. The reception of specimens to close March 1st, 1850. The Committee are, Mr. C. C. Williams and Dr. J. D. White of Philadelphla, and Dr. Ely Parry of Lancaster, Pa."

We do not expect that the manufacturers of porcelain teeth will be excited to emulation so much by the value of the premium offered by the Society, as by the value which they will place upon the award of preference. We hope a spirit of emulation may be excited among them, and that some valuable improvements in the manufacture of teeth may result from it. We do not believe that this branch of our art has yet arrived at perfection, and notwithstanding the many and great improvements that have been made in it during the last twenty years, we expect it will continue to advance, until porcelain teeth shall be made to resemble so nearly the natural organs, as, when placed in the mouth, to elude detection even from the closest observer.—*Balt. Ed.*

